# The sweet and the bitter sides of galectin-1 in immunity: its role in immune cell functions, apoptosis, and immunotherapies for cancer with a focus on T cells

**DOI:** 10.1007/s00281-025-01047-8

**Published:** 2025-04-03

**Authors:** Julianna Novák, Tamás Takács, Álmos Tilajka, Loretta László, Orsolya Oravecz, Emese Farkas, Nándor Gábor Than, László Buday, Andrea Balogh, Virág Vas

**Affiliations:** 1 Signal Transduction and Functional Genomics Research Group, Institute of Molecular Life Sciences, HUN-REN Research Centre for Natural Sciences, Budapest, 1117 Hungary; 2 Doctoral School of Biology, Institute of Biology, Eötvös Loránd University, Budapest, 1117 Hungary; 3 Systems Biology of Reproduction Research Group, Institute of Molecular Life Sciences, HUN-REN Research Centre for Natural Sciences, Budapest, 1117 Hungary; 4 Károly Rácz Conservative Medicine Division, Doctoral College, Semmelweis University, Budapest, 1091 Hungary; 5 Department of Obstetrics and Gynecology, Semmelweis University, Budapest, 1088 Hungary; 6 Department of Molecular Biology, Semmelweis University, Budapest, 1094 Hungary

**Keywords:** Apoptosis, Cancer immune evasion, Galectin-1, Immune checkpoint inhibitors, Immunotherapy, T lymphocytes

## Abstract

Galectin-1 (Gal-1), a member of the β-galactoside-binding soluble lectin family, is a double-edged sword in immunity. On one hand, it plays a crucial role in regulating diverse immune cell functions, including the apoptosis of activated T cells. These processes are key in resolving inflammation and preventing autoimmune diseases. On the other hand, Gal-1 has significant implications in cancer, where tumor cells and the tumor microenvironment (TME) (e.g., tumor-associated fibroblasts, myeloid-derived suppressor cells) secrete Gal-1 to evade immune surveillance and promote cancer cell growth. Within the TME, Gal-1 enhances the differentiation of tolerogenic dendritic cells, induces the apoptosis of effector T cells, and enhances the proliferation of regulatory T cells, collectively facilitating tumor immune escape. Therefore, targeting Gal-1 holds the potential to boost anti-tumor immunity and improve the efficacy of cancer immunotherapy. This review provides insights into the intricate role of Gal-1 in immune cell regulation, with an emphasis on T cells, and elucidates how tumors exploit Gal-1 for immune evasion and growth. Furthermore, we discuss the potential of Gal-1 as a therapeutic target to augment current immunotherapies across various cancer types.

**Supplementary Information:**

The online version contains supplementary material available at 10.1007/s00281-025-01047-8.

## Introduction to galectin-1

Carbohydrate-binding galectins are expressed in almost every living organism, from the simplest invertebrates to the most advanced vertebrates [1], and have versatile functions in these species. Taken together all gene copy-variants, in mammals, 22 group members have been identified so far (galectin-1, -2, -3, -4, -5, -6, -7, -7b, -8, -9, -9b, -9c, -10, -11, -12, -13, -14, -15, -16, -17, -19, -20) [2–5], out of which 16 are expressed in human tissues (all except galectin-5, -6, -11, -15, -19, -20). These proteins have highly conserved regions called carbohydrate recognition domains (CRD), which enable them to bind to carbohydrate moieties containing β-galactose [1]. Based on the CRD number and structural organization, galectins are classified into three major groups [1]: (1) proto-type galectins possess a single CRD and can homodimerize through covalent (disulfide) bonds (in the case of Gal-13 [6] but mostly through non-covalent interactions (hydrogen bonding, van der Waals and electrostatic forces), which is usually important for the cross-linking of glycoprotein receptors and cellular effects, such as found for gal-1 and gal-10; (2) tandem-repeat-type galectins contain two different CRDs, allowing them to interact with different target molecules at the same time; and (3) the only chimeric galectin (galectin-3) is unique as it possesses an additional non-CRD alongside its CRD. Most galectins can oligomerize and display a cross-linker function, which is key to the formation of galectin-glycan lattices on cell surfaces that regulate glycoprotein organization and signaling [7]. Recent studies also indicate that galectins may form heterodimers and -oligomers with functional significance, especially when they are temporally and spatially co-expressed, similar to chemokines [8]; however, further in vitro and in vivo experiments are needed to make the hypothesis more solid and widely accepted. Of importance, the diverse binding capabilities of galectins complicate studying their effects on cellular functions, as the proteins’ behavior can vary dramatically depending on various factors, such as their source and the surrounding microenvironment. These challenges highlight the limitations of in vitro and in vivo research, where results are not necessarily consistent due to neglecting the dynamics of endogenous galectins and the interplay of their intracellular and extracellular actions, which will be detailed later. Being aware of this, we present a comprehensive overview of galectin-1 (Gal-1), the most extensively studied member of the galectin family, with a specific focus on its immunological functions, its involvement in tumor biology, and its potential as a therapeutic target in tumor immunotherapy.

### The structure and ligand binding of galectin-1

**Fig. 1:**
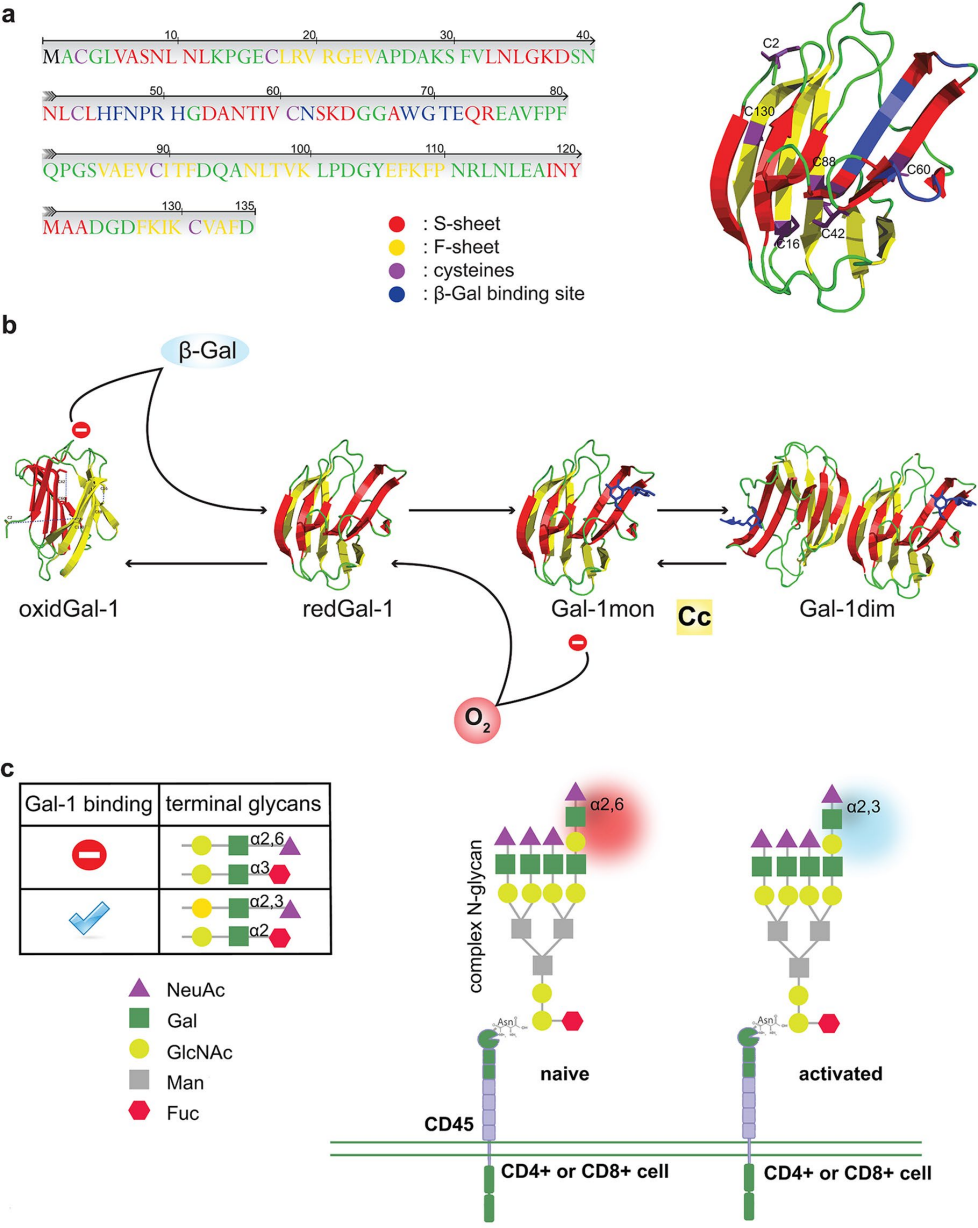
Structure and carbohydrate-binding of galectin-1. **(a)** The amino-acid sequence and the 3D structure of Gal-1 are depicted, indicating the cysteines (purple), the ligand binding site (blue), and the β-strands (red and yellow). **(b)** Gal-1 oxidation, dimerization, and ligand binding influence each other. Firstly, an oxidative environment induces Gal-1 monomer (redGal-1) oxidation, but ligand binding (β-Gal) partially protects Gal-1 from oxidation. Secondly, oxidized Gal-1 (oxidGal-1) binds fewer ligands due to conformational changes. Thirdly, Gal-1 concentration (Cc) itself highly affects the kinetics of association and dissociation. The functionally active Gal-1 dimer depends on these three factors (O_2_, ligand, Cc). **(c)** Terminal modifications highly influence Gal-1/glycan interaction: α2,6-sialylated and α3-fucosylated residues reduce, while α3-sialylated and α2-fucosylated enhance (+) Gal-1 binding to its glycoprotein interacting partners, e.g., to CD45 with different glycosylation states on naive or activated peripheral T cells.

Gal-1 is a proto-type galectin, the earliest identified member of this family [9]. It is a 14.5 kDa compact globular protein consisting of 135 amino acids (Fig. 1a). The structure of Gal-1 is characterized by two β-folds (S- and F-sheets) facing each other, creating a β-sandwich, in which the carbohydrate-binding region is located [9] (Fig. 1a). It can dimerize *via* amino- and carboxy-terminal hydrophobic amino acids, opposite the carbohydrate-binding pocket. The dimerization is reversible, and the physiological dissociation constant (Kd) is ~ 1–7 µM [10]. At concentrations ≤ 1 µM, Gal-1 exhibits minimal immunomodulatory signaling activity. The association/dissociation kinetics are influenced by the concentration of Gal-1 and the presence of ligands: at lower concentrations and in the absence of ligands, the rate of dissociation increases [11]. Furthermore, Gal-1 biological activity is correlated with homodimer-mediated cross-linking of cell-surface glycoproteins into lattices [12]. In accordance, a stable homodimer state increased its biological activity in various in vitro assays [13–15].

The structure of Gal-1 is highly sensitive to the redox state of its microenvironment. Within its amino acid sequence, there are six cysteines (C2, C16, C42, C60, C88, and C130) (Fig. 1a), which can be oxidized easily to form intramolecular disulfide bridges [16], altering the spatial structure of the protein [17]. Oxidized Gal-1 loses its ability to bind to carbohydrates due to conformational changes, particularly the formation of a disulfide bridge between C16 and C88, which alters amino acid interactions in the carbohydrate-binding motif [16]. In a pathophysiological context, for instance, circulating Gal-1 is oxidized in systemic lupus erythematosus and is not recognized by V-set and transmembrane domain-containing 1 (VSTM1), to which it binds in a carbohydrate-independent manner. This results in a decrease in neutrophil viability and an increase in intracellular reactive oxygen species levels [18].

Interestingly, the balance between the monomeric and dimeric forms of Gal-1 may also affect its susceptibility to oxidation. For example, Gal-1 with C2S and V5D mutations, which prevent dimerization, is more susceptible to oxidation [19]. Moreover, ligand binding can partially protect Gal-1 from oxidation [13] and reduce dissociation [11]. Based on the data above, a possible model can be proposed to explain the interplay between oxidation, dimerization, and ligand binding, as summarized in Fig. 1b. Additional findings add complexity to this model. For instance, oxidized Gal-1 has been shown to be a potent inducer of inflammatory cytokines in macrophages, vascular smooth muscle cells, and fibroblasts, mediated *via* the mitogen-activated kinase (MAPK) pathway [20]. Furthermore, oxidized Gal-1 can protect MOLT-4 lymphoblastic leukemia cells from H_2_O_2_-induced apoptosis [21], a function of potential relevance to infections, inflammation, and cancer. Collectively, these results underscore the structural and functional complexity of Gal-1, demonstrating that while its dimeric/reduced form is required for many of its biological functions, its oxidized form retains specific roles depending on the cell type and the (glyco)-receptors involved.

The binding preferences of galectins to specific glycan modifications play a crucial role in determining their interactions with glycosylated molecules [22, 23]. Gal-1 typically binds to the disaccharide N-acetyllactosamine, which is present in both N-glycans (attached to asparagine residues of proteins) and O-glycans (linked to serine/threonine residues of proteins) [24]. The binding affinity of Gal-1 increases when it encounters complex polysaccharides with branching, antenna-like structures. Apart from the arrangement of carbohydrate molecules, the structure of the terminal glycan also impacts Gal-1-glycan interaction. Specifically, Gal-1 exhibits stronger binding to α2,3-sialylated and α2-fucosylated lactosamine residues, which enhances its affinity. Conversely, α2,6-sialylated and α3-fucosylated terminal glycans impair Gal-1-glycan interaction [25] (Fig. 1c). This knowledge is indispensable for the rational design of small-molecule lactose/N-acetyllactosamine-based carbohydrate inhibitors of Gal-1 for treating cancer and other pathological conditions in the future [26].

### The expression of galectin-1

**Fig. 2:**
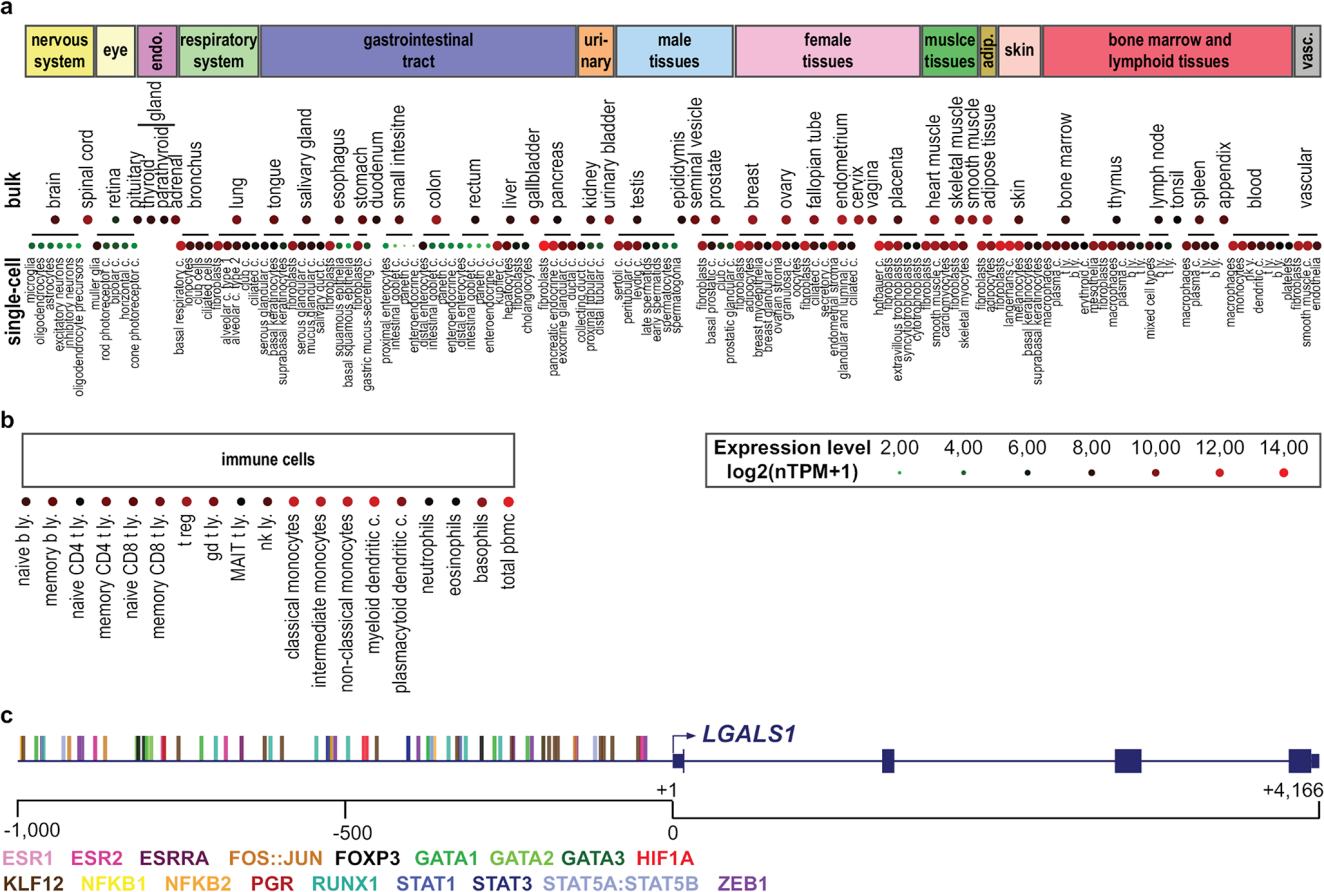
Expression of *LGALS1*, encoding galectin-1, and its transcriptional regulation. The transcript expression level of *LGALS1* in humans, derived from bulk-tissue and single-cell RNA-seq **(a)**, as well as RNA-seq of immune cells **(b)**, is depicted on the heatmaps. Data were downloaded from the Human Protein Atlas on 13-05-2024 (consensus bulk RNA-seq data of HPA and GTEx projects, single-cell RNA-seq data from various external sources, and immune cell gene data [32, 33], and visualized after log-transformation [log_2_(nTPM + 1)], using the TBTools-II platform [34]. Expression level of the same cell type per tissue was averaged. Not all cell types were visualized. Table S1 contains the full datasets. **(c) ***LGALS1* gene and 1000 bp 5’ upstream region with selected transcription factors (TFs) are illustrated (adapted from the USCS Genome Browser [35]. The complete list of TFs can be found in Table S2. The list is a cross-section of datasets from JASPAR 2024 [36] and TFLink [37] open-access databases downloaded on 04-04-2024, with a minimum score of 240. Adip, adipose; c, cells; endo, endocrine; ly, lymphocytes; MAIT, mucosal-associated invariant T; PBMC, peripheral blood mononuclear cells; RNA-seq, RNA sequencing; nTPM, normalized transcripts per million; vasc, vasculature.

Gal-1 has the broadest expression among galectins both in humans (Fig. 2a, Table S1) and mice, highlighting its pivotal role in numerous biological processes. The expression of Gal-1 can be modulated by a variety of factors, such as differentiation and activation signals [27], hypoxia [28], cytokines [29], and hormones [30]. *LGALS1*, the gene coding for Gal-1, expression is also reflected in immune cells’ activation and differentiation status, as memory T and B cells have much higher expression than the naive ones [22, 27]. Among T cells, regulatory T cells (Tregs) bear the highest *LGALS1* expression (Fig. 2b) [31]. To mention a few significant transcription factors (TFs) regulating *LGALS1* expression, the promoter region of *LGALS1* contains hypoxia-response elements (HREs) for hypoxia-inducible factor 1-alpha (HIF-1α) binding [28], binding sites for nuclear hormone receptors [e.g., estrogen receptor 1 (ESR1), ESR2, estrogen-related receptor alpha (ERRα), progesterone receptor (PGR) [32], and other key TFs like signal transducer and activator of transcription (STAT) proteins (e.g., STAT3) [33], FOS/JUN proto-oncogene, and regulators of cell differentiation, including forkhead box P3 (FOXP3), a master regulator of Treg differentiation (Fig. 2c, Table S2). For instance, among several vascular endothelial growth factor 1 (VEGFR1) ligands induced by hypoxia, placental growth factor (PGF) alone upregulated *LGALS1* expression in an activator protein 1 (AP-1)-dependent manner [28]. However, we have to keep in mind that the regulation of *LGALS1* expression varies depending on the cell type. Indeed, tumor necrosis alpha (TNF-α) and interferon gamma (IFN-γ) have been reported to induce Gal-1 expression in endothelial cells [34] but not in mesenchymal stem cells (MSCs) [35], which indicates that cytokine-induced changes in Gal-1 expression/secretion may act as a mechanism to either potentiate or hamper the immune response, depending on the context [29]. However, MSCs already express Gal-1 abundantly, and they can be drawn to the site of inflammation by inflammatory stimuli. There, they aid in immunological homeostasis and tissue repair, both of which depend on Gal-1 [36, 37].

Although the precise regulation of Gal-1 expression remains incompletely understood, changes in its expression have been observed in a range of pathological conditions, including inflammatory diseases (e.g., tissue injuries, fibrosis, and autoimmune disorders) and cancers [38]. For instance, higher expression of AP-1 subunits FOS and JUN is associated with poor prognosis in several tumors [39], likely contributing to elevated *LGALS1* expression in these tumors. Accordingly, in classical Hodgkin lymphoma (cHL), Reed-Sternberg cells have been demonstrated in vitro to selectively overexpress Gal-1 *via* an AP-1-driven enhancer [40]. Later, immunohistochemistry experiments established a functional AP-1 signature that includes Gal-1 expression in cHL and anaplastic large-cell lymphoma, suggesting a common mechanism of tumor-induced immunotolerance in these malignancies [41]. Of note, epigenetic and post-transcriptional modifications may also contribute to dysregulated *LGALS1* expression in pathological conditions [42–45].

The broad tissue expression of *LGALS1* is reflected in its particularly high levels in the blood, where it is the most abundant galectin [46, 47]. Galectins are synthesized in the cytoplasm on free ribosomes and exported to the extracellular milieu by non-classical secretion *via* direct transport or packed into extracellular vesicles (EVs) [48]. This pathway is especially significant because the biological activity of galectins may be influenced by the type of secretion and how they reach their target cells. Consequently, total, free, or EV-bound Gal-1 levels could have diagnostic value in several pathological events, including inflammation, autoimmune diseases, and tumors [49–52].

## Main physiological functions of galectin-1

Gal-1 modulates signaling pathways by cross-linking glycosylated receptors [e.g., CD7, CD43, CD45, fibroblast growth factor receptor (FGFR)] on the cell surface [5, 53]. Gal-1 is also involved in regulating cell adhesion and migration by binding to integrins (such as α1β1, α5β1, α7β1, α2, α3, αv, and αIIbβ3) found in the plasma membrane of target cells (including trophoblasts and tumor cells) [54] and extracellular matrix (ECM) components like laminin and fibronectin [55]. Of note, intracellular Gal-1 regulates various cellular processes, such as H-ras nanoclustering/signaling and pre-mRNA-splicing, which is beyond the scope of our review and has been comprehensively reviewed [56, 57]. Thereafter, we will only mention intracellular Gal-1 if it is contextually relevant. One particularly intriguing aspect of the extracellular−intracellular Gal-1 axis is the dynamic reciprocity between the glycan microenvironment and nuclear Gal-1 levels. On one hand, the abundant presence of N-acetyllactosamine epitopes in the extracellular environment has been found to act as a molecular sink, trapping Gal-1 outside epithelial cells. Conversely, when Gal-1 was unable to bind extracellular glycan ligands (mimicked with a mutant Gal-1), a shift occurred, resulting in a higher nuclear abundance of Gal-1, which promoted epithelial invasiveness [58]. These findings likely extend beyond epithelial cells, suggesting that Gal-1 can directly transmit glycan-encoded information from its surroundings to the nucleus, with potential relevance for its physiological and pathological roles, including immunity and cancer.

**Fig. 3:**
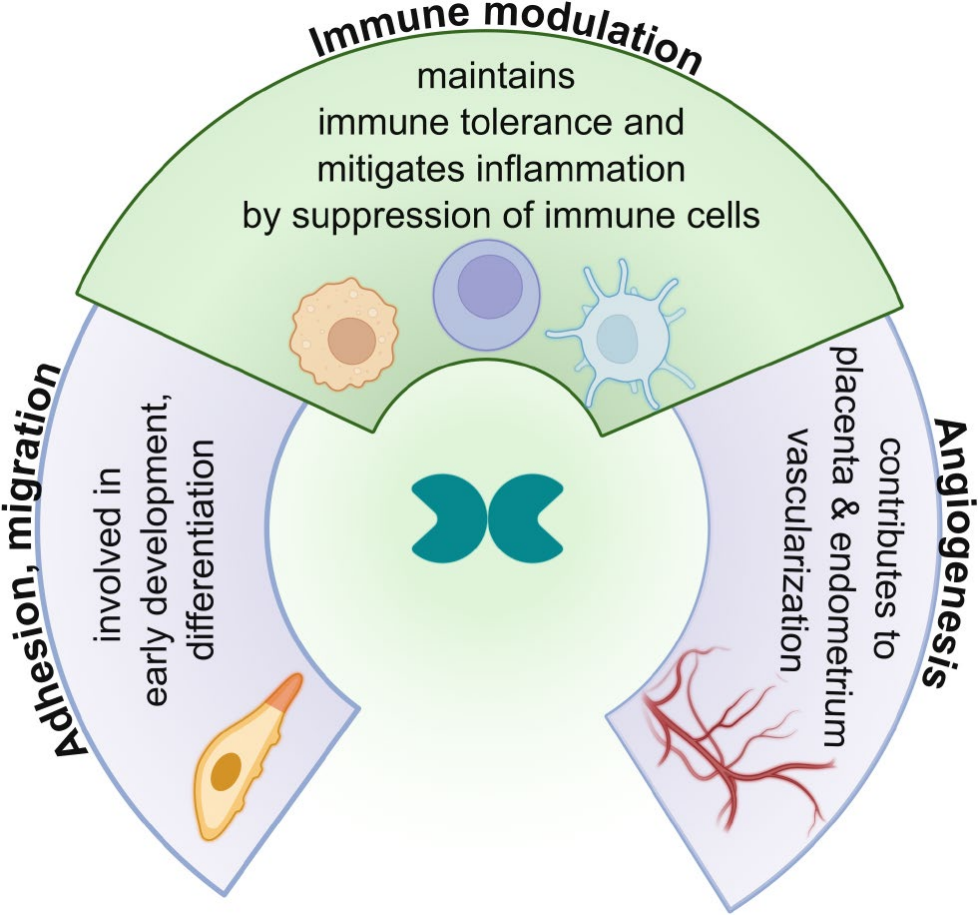
Main physiological functions of galectin-1. Gal-1 is involved in the differentiation and development of embryonic and adult organs by regulating cell adhesion and migration processes, the maintenance of immune tolerance by immune cell suppression in immune-privileged organs and inflammation resolution, and angiogenesis in various tissues, e.g., the placenta and endometrium.

Interestingly, homozygous knockout mice lacking Gal-1 do not exhibit dramatic phenotypic differences compared to wild-type mice. However, these knockout animals do show certain developmental delays, such as in early olfactory nerve and muscle tissue development [59]. Additionally, they exhibit behavioral differences like reduced social dominance, delayed initiation of movement, and heightened anxiety [60]. These mice are also more susceptible to experimentally induced autoimmune encephalitis, rheumatoid arthritis, and stress-related miscarriage [61–64]. These phenotypes draw attention to the most important physiological functions of Gal-1; this lectin participates in the differentiation and development of organs, maintains immune tolerance by regulating immune cells, and promotes blood vessel formation in several tissues [65, 66], e.g., decidua and placenta (Fig. 3).

### Dual effect of galectin-1 on immune cells

Gal-1 is crucial for hematopoiesis, as reflected by its high expression in the bone marrow by MSCs and endothelial cells. Recombinant Gal-1 concentration-dependently influences the fate of hematopoietic stem cells (HSCs). It increases the formation of granulocyte-macrophage (and erythroid colonies) at low concentrations in a lactose-inhibitable fashion, but at high concentrations, it reduces the growth of the committed blood-forming progenitor cells and lineage-negative HSCs, which could not be blocked by lactose [67]. However, Gal-1 inhibits hematopoietic progenitor cell-, granulocyte-, and monocyte trafficking to the periphery, so it may contribute to the attenuation of acute and chronic inflammation [68]. Gal-1 also supports B lymphocyte maturation by serving as the ligand for pre-B cell receptor (pre-BCR), promoting receptor clustering and integrin re-localization at the synaptic connection between stromal cells and pre-B cells, thus amplifying activation signals for pre-B cell expansion [69, 70]. Thymocyte development is also regulated by Gal-1, as will be discussed later.

Gal-1 is a key player in mitigating inflammatory processes and developing immune tolerance by affecting virtually all immune cells [71]. Accordingly, it has enhanced expression at the site of inflammation during the resolution phase and at the sites of central and peripheral tolerance [22]. Moreover, Gal-1 is highly expressed in immune-privileged areas of the body, such as the brain, reproductive organs, decidualized endometrium, and the placenta [72–76]. Relatedly, Gal-1 is one of the crucial factors responsible for the establishment and maintenance of immune tolerance at the maternal-fetal interface, acting directly on dendritic cells (DCs) and natural killer (NK) cells and indirectly on Tregs [62, 77]. While data is limited, Gal-1 can also positively regulate some immune cell functions depending on factors such as concentration, the abundance of glycoepitopes on cell surfaces and extracellular milieu, and the mode of action. Among immune cells, Gal-1 is mainly produced by tolerogenic DCs [78], activated and memory T and B cells [27, 79], activated and inflammatory macrophages [80], Tregs [81], γδ T cells [82] (Fig. 2b), and decidual NK cells [83], underscoring that activation and differentiation status determine Gal-1 expression in these cells; furthermore, it complicates the discrimination between immune effects of Gal-1 when intracellular or secreted.

In macrophages, exogenous administration of recombinant Gal-1 reduces inflammatory responses by inhibiting the release of arachidonic acid and prostaglandin E2, suppressing the activity of inducible nitric oxide synthase (iNOS), and decreasing the production of the pro-inflammatory cytokine interleukin (IL)-12 [84, 85]. Gal-1 also promotes macrophage polarization towards the M2 phenotype, which is associated with anti-inflammatory responses and tissue regeneration by regulating L-arginine metabolism [85]. Accordingly, during the resolution phase of inflammation, a Gal-1−IFN-β axis was identified as involved in reprogramming reparative/phagocytic/CD11b^high^ macrophages to the pro-resolving/CD11b^low^ phenotype [86]. Moreover, recombinant Gal-1 has been found to disrupt the migration of monocytes by reducing the expression of CD49d (α4 integrin) [87], the activation, chemotaxis, and migration of neutrophil granulocytes [88], and the migration and degranulation of eosinophil granulocytes during acute inflammation [89]. On the contrary, it has been evidenced by in vitro and in vivo experiments that Gal-1 treatment can promote neutrophil migration potentially through binding to glycoepitopes on CD43 and activating the p38 MAPK pathway. This finding suggests that depending on the microenvironment, Gal-1 can act either as a chemoattractant or as a negative regulator of migration under acute inflammatory conditions [90].

In DCs, Gal-1 has a concentration-dependent dual regulatory role. At high Gal-1 concentrations (> 20 µM), the pro-inflammatory functions of DCs are induced, including increased maturation and migration. However, at low concentrations (< 20 µM), Gal-1 acts as an immunosuppressive factor by inducing a tolerogenic phenotype in DCs and inhibiting their maturation and antigen-presenting capacity [91]. These tolerogenic DCs produce IL-27 and induce the secretion of IL-10 in T cells, promoting the expansion of Tregs [78, 92]. Additionally, ECM-bound Gal-1 selectively inhibits the migration of immunogenic but not tolerogenic DCs by clustering CD43 *via* binding to core 2 O-glycans, more abundant on the surface of immunogenic DCs [93].

In the lymphoid lineage, recombinant Gal-1 has been found to promote immunoglobulin production during plasma cell differentiation [94]. Furthermore, in *Lgals1*^*−/−*^ mice, cytokine expression and suppressive capacity of transitional regulatory B cells have been reduced; however, the authors could not conclude whether Gal-1 plays its role extracellularly or intracellularly [95].

Impaired immune cell functions associated with Gal-1 deficiency or blockade have been observed in rodent models of inflammatory autoimmune diseases, such as systemic lupus erythematosus, collagen-induced rheumatoid arthritis, and non-obese type 1 autoimmune diabetes [96]. All these findings show that Gal-1 can act in both autocrine and paracrine manners to control the functions of immune cells and underscore its key role in immune tolerance and autoimmunity.

## The role of galectin-1 in T cell regulation

**Fig. 4:**
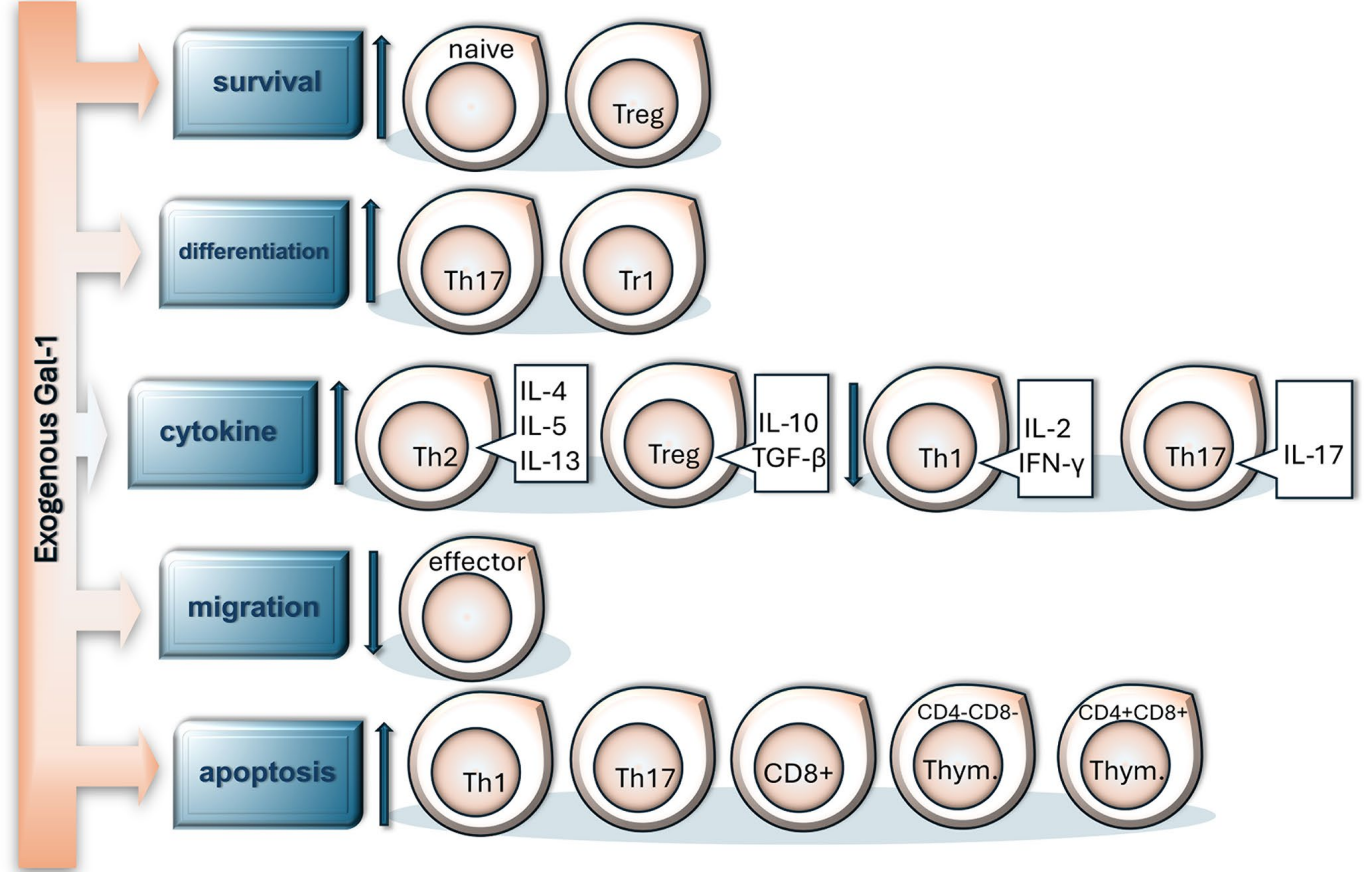
Gal-1 regulates T cell subpopulations at multiple levels. Gal-1 (derived from, e.g., stromal cells, activated endothelial cells, fibroblasts, macrophages, and dendritic cells) has various effects on T cells. It promotes naive and regulatory T cell survival, enhances the differentiation of Th17 and Tr1 cells, and boosts the production of anti-inflammatory cytokines in Th2 and Treg cells, but decreases the production of IL-2, IFN-γ, and IL-17 in Th1 and Th17 cells. It impairs effector T cell migration and induces the apoptosis of Th1, Th17, and Tc lymphocytes, as well as double-negative and double-positive thymocytes. IFN, interferon; IL, interleukin.

As mentioned earlier, Gal-1 has a versatile effect on all immune cells, although it has the most profound impact on T lymphocytes; it influences these cells’ survival, differentiation, adhesion, migration, and cytokine production, and induces their apoptosis [97] (Fig. 4). Studies have demonstrated that Gal-1, secreted by stromal cells in lymph nodes, promotes the survival of naive T cells without enhancing their proliferative capacity [98, 99]. In addition, this lectin also plays a role in the differentiation of Th and Treg subsets and their cytokine production. Recombinant gal-1 has been found to inhibit IL-17 and IFN-γ production as well as Retinoic acid receptor-related orphan receptor C2 (RORC2) TF expression in differentiated Th17 cells through its carbohydrate-dependent interaction with CD69 [100]. Meanwhile, the stable dimeric form of Gal-1 binds directly to CD45 on the cell surface of unpolarized activated Th cells and induces IL-10 expression *via* STAT3 phosphorylation. These cells then differentiate into FOXP3^−^ type 1 regulatory (Tr1)-like cells and effectively suppress the proliferation of other T cells [101]. Indirectly, Gal-1-induced tolerogenic DCs secrete IL-27, which can further drive Tr1 cell differentiation and IL-10 production. These Gal-1-generated Tr1 cells have been shown to efficiently suppress inflammation in mouse autoimmune models and promote tumor growth and immune evasion in syngeneic mouse cancer models [78]. Administration of recombinant Gal-1 or co-culture with Gal-1-expressing cells (e.g., tumor cells and trophoblasts) leads to the expansion of classical FOXP3^+^ CD25^+^ Tregs as well [102].

Gal-1 can modulate the production of various cytokines in T cells depending on the subpopulation. Studies have demonstrated that low concentrations of recombinant Gal-1 directly suppress the production of pro-inflammatory Th1 cytokines (IFN-γ and IL-2) without affecting T cell viability [103], while selectively inducing the apoptosis of Th1 and Th17 cells, responsible for pro-inflammatory cytokine production [81]. On the other hand, exogenously added Gal-1 promotes the production of cytokines – IL-4, IL-5, and IL-13 – in Th2 cells [104]. Additionally, several reports have confirmed that recombinant Gal-1 induces the production of IL-10 in inactivated and activated Th, Tc, and Treg cells, both in vitro and in vivo [13, 101, 105].

Cell surface carbohydrates and specific glycan-binding proteins play crucial roles in guiding lymphocytes to secondary lymphoid organs, inflamed tissues, or tumors [106]. In line with its anti-inflammatory effects, Gal-1 has been shown to inhibit adhesion and transendothelial migration of T cells. Mechanistically, endothelial cell-derived Gal-1 clusters CD43 on the surface of T cells and, similarly to DCs and opposite to neutrophils, hinders their transendothelial migration [107]. Furthermore, Gal-1 negatively impacts the attachment of activated T cells to glycoproteins in the ECM by inhibiting the F-actin re-organization [108].

T cells are not only targets for Gal-1 binding to their cell surface but also express it endogenously, where it functions in autocrine and paracrine manners. According to Chung et al., changes in glycosyltransferase and Gal-1 expression patterns in activated and memory T cells suggest that endogenous Gal-1 may inhibit proliferation and induce apoptosis in T cells during the resolution phase of Ag-specific immune responses [109]. Supporting this, central memory Tc cell development as well as IFN-γ secretion by effector Tc cells was increased in chimeric mice (with *Lgals1*^−/−^ bone marrow cells) in contact hypersensitivity without affecting the suppressive capacity of Tregs [110]. Furthermore, Gal-1-deficient Tc cells divide more in response to T cell receptor (TCR) stimulation, with fewer dividing cells undergoing apoptosis since they lack Gal-1 as a negative regulator of TCR binding, signal transduction, and burst size [111]. However, Deák et al. proposed that although peripheral human and mouse T cells start to express Gal-1 upon polyclonal activation, they do not secrete it. As such, *de novo* synthesized Gal-1 does not directly induce T cell death in an autocrine fashion but sensitizes T cells to both soluble and membrane-bound extracellular Gal-1 [105]. Additionally, *Lgals1*^*−/−*^ Tc cells expanded at a higher frequency and were capable of proper degranulation upon specific stimulation, but they had reduced cytotoxic ability. Mechanistically, Gal-1 prevented the internalization of FasL, thereby prolonging Fas–FasL interactions at the Tc–target cell interface [112]. Collectively, the role of endogenous Gal-1 produced by activated and memory T cells is still elusive and needs further investigation.

Another aspect of the Gal-1-exerted T cell regulation is linked to Treg population dynamics. Transcriptomic and proteomic studies that focused on identifying immunosuppressive molecules specific to CD4^+^ CD25^+^ Tregs revealed that Gal-1 is highly expressed in both mice and humans. Garin et al. conducted experiments where they blocked Treg activity using an anti-Gal-1 neutralizing antibody. They observed that although the number of Treg cells in Gal-1 knockout mice was similar to that in wild-type mice, the suppressive capacity was significantly reduced when evaluated ex vivo [81]. Wang et al. further elucidated the mechanism by which Tregs inhibit effector T cells and found that the interaction between Gal-1 and GM1 ganglioside on the surface of effector cells is a critical factor [113]. Additional studies using in vivo models of autoimmune eye inflammation [109] and stress-induced pregnancy loss [49] have found that therapeutic administration of Gal-1 restored T cell tolerance, leading to a significant increase in the number of IL-10- and TGF-β-producing CD4^+^CD25^+^ Treg cells [114]. These results show that the enhancement of IL-10-producing Treg functions, which may be associated with Gal-1, holds promise as a therapeutic target for preventing organ transplant rejection [115], improving tolerance between the mother and fetus, and reducing inflammatory reactions [116]. These findings also point out that a weakened Gal-1-mediated response may lead to a stronger anti-tumor immune response, which we discuss later.

## Galectin-1 induces T cell apoptosis

In the thymus, the development of T cells involves a critical checkpoint mediated by TCR signaling. Thymocytes with high-affinity TCRs for self-peptide − MHC complexes undergo apoptosis (negative selection), while those with low-affinity TCRs survive and differentiate into mature T cells (positive selection) [117]. In vitro experiments have demonstrated that Gal-1, expressed by thymic epithelial cells, promotes apoptosis of both double-negative and double-positive immature thymocytes [118]. Endogenous Gal-1 influences TCR-mediated negative selection by inducing rapid and transient activation of extracellular signal-regulated kinase (ERK) while inhibiting ERK activity during positive selection. Thus, Gal-1 can modulate TCR signaling in thymocytes undergoing negative and positive selection by exerting dual regulatory effects [119]. In the latter study, although not discussed, thymocyte-derived Gal-1 likely exerted its effects through binding to cell surface ligands.

In the periphery, Gal-1 induces the apoptosis of activated T cells, presumably through distinct mechanisms than in thymocytes. This lectin specifically triggers cell death in Th1 and Th17 cells but does not induce the apoptosis of the Th2 cell subpopulation (Fig. 4), because of the distinct glycosylation patterns on the cell surfaces of these individual T cell subsets [63]. ECM glycoproteins, such as laminin, fibronectin, and vitronectin, play multiple roles in mediating Gal-1-induced T cell apoptosis by interacting with Gal-1, preserving its carbohydrate-binding activity, and concentrating it to increase the fraction of homodimer Gal-1. Furthermore, the ECM presents Gal-1 to the T cell surface in a 2D array rather than in a 3D space as when T cells encounter soluble Gal-1; this type of presentation may facilitate the interaction of Gal-1 with large glycoproteins like CD43 and CD45, which form a glycocalyx surrounding T cells, enhancing the rate and/or duration of Gal-1 binding [120].

The literature on Gal-1-induced T cell apoptosis is contradictory, likely due to several factors. Oxidation of Gal-1, for instance, triggers structural changes that reduce its carbohydrate-binding capability, resulting in compromised T cell apoptosis [114]. The glycosylation profile of T cells, which changes during activation and differentiation, also plays a critical role. Gal-1 selectively binds to specific N- and O-glycosylated structures, present only when certain glycosidase and glycosyltransferase enzymes are expressed [121]. The sensitivity of T cells to Gal-1-induced apoptosis depends on the ratios of sialyltransferases (ST6GAL1, ST3GAL6) to neuraminidase 1 (NEU1) [122]. Th1, Th17, and activated cytotoxic T cells (Tc) are susceptible to Gal-1-induced apoptosis, while Th2 and naive T cells, which express inhibitory α2,6 sialylated glycans, are resistant. Furthermore, human decidual T cells have a distinct glycophenotype due to the expression of core 2 β-1,6-N-acetylglucosaminyltransferase (C2GNT), allowing Gal-1 secreted by NK cells and other cells of the decidua to induce T cell apoptosis [123].

So far, CD2, CD3, CD4, CD7, CD43, CD45, CD69, and CD95 glycoproteins and the glycolipid GM1 have been identified as Gal-1 interaction partners in the plasma membrane of T cells [5, 124]. Early studies suggested that CD45 plays a role in transmitting Gal-1-induced apoptotic signals [125], although subsequent research indicated that while Gal-1 binds to CD45, this interaction is not essential for T cell death [126]. Instead, CD45 can both enhance and inhibit Gal-1-mediated apoptosis, depending on the context [124]. These discrepancies may be attributed to the differential expression of CD45 isoforms, which exhibit distinct N- and O-glycosylation patterns in their extracellular domains across various T cell subsets. These isoform differences are closely tied to T cell maturation and activation states, influencing functional outcomes [127].

Gal-1 has been found not to act through a single receptor. Studies by Chung et al. suggest that Gal-1 modulates TCR signaling by limiting membrane microdomain reorganization and sustained signal transduction. Gal-1 cross-links lipid raft-associated components (GM1, CD4), raft-translocable proteins (CD3), and non-raft constituents (CD43, CD45), thereby restricting lipid raft cluster sizes within immunological synapses and allowing TCR-induced functions that require only partial TCR signal transduction [109]. This is further supported by observations that CD45 co-localizes with CD3, and CD7 with CD43, within distinct microdomains upon Gal-1 binding, exclusively in apoptotic T cells [128]. These findings align with the galectin-glycan lattice theory, which posits that galectins form oligomeric networks capable of decoding glycan information on the cell surface [23].

The ganglioside GM1, a common lipid raft component involved in various T cell functions, including TCR-mediated activation, is a crucial Gal-1 interaction partner [129]. GM1 levels significantly increase upon T cell activation and differentiation [130]. GM1 and CD7, but not CD2, CD3, CD43, or CD45, are internalized into T cells alongside Gal-1, with strong co-localization suggesting their involvement in Gal-1 sorting into the Golgi apparatus [131]. Moreover, cross-linking GM1 with cholera toxin subunit B (CtxB) or Gal-1 has been shown to induce immunosuppression in several studies. For instance, GM1-deficient effector T cells are less susceptible to suppression by Tregs, and CtxB or Gal-1 treatment more effectively inhibits the proliferation of control effector T cells compared to GM1-deficient ones [132]. In line with this, Gal-1-induced apoptosis is diminished in T cells with low GM1 levels [133]. Moreover, cross-linking GM1 with Gal-1 or CtxB has been shown to inhibit T cell proliferation and ameliorate symptoms in an experimental autoimmune encephalitis mouse model. This model demonstrated that Gal-1 derived from Tregs and GM1 in effector T cells are both critical for this suppression, as Gal-1-neutralizing antibodies blocked Treg-mediated suppression, and Gal-1-deficient effector T cells exhibited impaired response to Treg suppression [113]. However, according to a novel study using model membranes, cross-linking does not result from sole interactions between GM1 and Gal-1, indicating that additional triggers are needed in a physiological context [134].

A third critical factor in T cell apoptosis is the concentration of Gal-1. The mechanism of T cell apoptosis has been extensively investigated using soluble recombinant Gal-1 at different concentrations in vitro, and the results have been inconsistent. Some studies suggest that Gal-1-mediated T cell apoptosis is the mitochondrial type of caspase-mediated cell death, whereas others indicate that mitochondrial depolarization occurs independently of caspase activation, involving B-cell lymphoma 2 (Bcl-2) downregulation, AP-1 TF activation, and endonuclease G nuclear translocation, causing DNA degradation [135]. Additionally, research by Stowell et al. has found that Gal-1 induces phosphatidylserine (PS) exposure to the outer leaflet of the plasma membrane without triggering apoptosis [136]. These conflicting results are most likely the result of differences in purification methods and the concentrations of Gal-1 used. Specifically, both low (1.8 µM) and high (18 µM) concentrations of recombinant Gal-1 induce PS exposure, lipid raft reorganization, and mitochondrial depolarization, but only at low Gal-1 concentrations activate Lck (p56Lck) and ZAP70 required for caspase activation [133, 135].

## Galectin-1 and T cell-based immunotherapies for cancer

### Galectin-1 in cancer formation

**Fig. 5:**
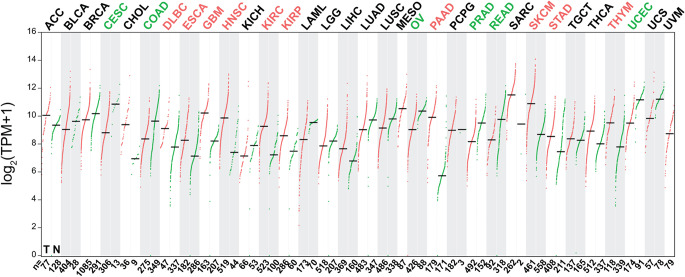
Comparison of galectin-1 gene expression in normal and tumor tissue samples. *LGALS1* expression was analyzed in the GEPIA2 database [139] with the following pipeline: we used the Expression DIY platform, where ANOVA was used as a statistical test, the|Log_2_FC| Cutoff was 1, the q-value Cutoff was 0.01, the Log scale was log_2_(TPM + 1), we matched TCGA to normal data with GTEx Green dots represent normal tissue (N) and red dots represent cancerous tissue (T) samples. In the upper row, tumors with significantly decreased Gal-1 expression are shown in green, and those with significantly increased expression are shown in red. ACC, adrenocortical carcinoma; BLCA, bladder urothelial carcinoma; BRCA, breast invasive carcinoma; CESC, cervical squamous cell carcinoma and endocervical adenocarcinoma; CHOL, cholangiocarcinoma; COAD, colon adenocarcinoma; DLBC, diffuse large B-cell lymphoma; ESCA, esophageal carcinoma; GBM, glioblastoma multiforme; HNSC, head and neck squamous cell carcinoma; KICH, kidney chromophobe; KIRC, kidney renal clear cell carcinoma; KIRP, kidney renal papillary cell carcinoma; LAML, acute myeloid leukemia; LGG, lower grade glioma; LIHC, liver hepatocellular carcinoma; LUAD, lung adenocarcinoma; LUSC, lung squamous cell carcinoma; MESO mesothelioma; OV, ovarian serous cystadenocarcinoma; PAAD, pancreatic adenocarcinoma; PCPG, pheochromocytoma and paraganglioma; PRAD, prostate adenocarcinoma; READ, rectum adenocarcinoma; SARC, sarcoma; SKCM, skin cutaneous melanoma; STAD, stomach adenocarcinoma; TGCT, testicular germ cell tumors; THCA, thyroid carcinoma; THYM, thymoma; TPM, transcripts per million; UCEC, uterine corpus endometrial carcinoma; UCS, uterine carcinosarcoma; UVM, uveal melanoma.

In contrast to its beneficial role in inflammatory and autoimmune disorders, Gal-1 contributes to the malignancy of several tumors by modulating multiple steps in tumor progression. Studies have shown that Gal-1 exhibits significant expression in several tumor types, including ovarian, colon, and liver cancers, where it is recognized as a negative prognostic marker [137]. Single-cell-level transcriptomics analysis of glycosylation-related genes and pathways also revealed that *LGALS1* is the most commonly upregulated transcript in cancer cells of the eight investigated tumor types, underscoring the significance of Gal-1 in cancer progression [138]. However, Gal-1 expression patterns in tumors are variable and cannot be explained by a simplified framework. Data from the GEPIA 2 database [139] indicate that Gal-1 expression is significantly decreased in six tumor types, increased in ten, and shows no significant changes in another seventeen tumor types compared to normal samples (Fig. 5). Expanding the database with more human samples will certainly improve the accuracy of these findings.

The direct effect of tumor-derived Gal-1 on T lymphocytes − involving enhanced T cell apoptosis, disturbed Th1/Th2 balance, and expansion of Tregs − is well-established [140]. Interestingly, Gal-1 was found to be present only in tumor-derived exosomes capable of inducing a suppressive phenotype of CD8^+^ T cells [141], demonstrating that both soluble and EV-packed Gal-1 are essential mediators of tumor immune escape. Nevertheless, Gal-1 is also abundantly present in the tumor microenvironment (TME) [142], primarily in fibroblasts, pericytes, and myeloid cells [138]. In this regard, the role of Gal-1 in fibroblast growth factor (FGF)−FGFR interaction and FGFR clustering [143, 144] is particularly important since Gal-1 expression is the highest in fibroblasts in almost all tissues, and FGFR amplification, mutations, fusions, or rearrangements can activate many pathways that lead to tumor development [145]. Both cancer cells and the surrounding stroma exploit the complex effects of Gal-1 to support several cancer hallmarks, including angiogenesis, invasion, tumor-associated inflammation, and immune evasion [146]. The molecular background of the tumor cell−TME interplay through Gal-1 was further elucidated in hepatocellular carcinoma (HCC), where HCC cells stimulated macrophages to secrete Gal-1 *via* toll-like receptor 2-mediated secretory autophagy, promoting HCC growth in mice and correlating with poor patient prognosis [147]. Exogenously added Gal-1 also induces changes in the gene expression profiles of M1- and M2-like macrophages by increasing the expression of e.g., indoleamine 2,3-dioxygenase (*IDO*), C-X-C motif chemokine ligand 5 (*CXCL5*), IL-1β, IL-4, and Janus kinase 3 (*JAK3*), all of which are involved in the regulation of tumor-associated macrophages (TAMs). A quantitative proteomics experiment further showed that Gal-1 polarizes M2-like macrophages toward a TAM-like phenotype by upregulating three prominent immunomodulatory proteins: the anti-inflammatory enzyme cis-aconitate decarboxylase (ACOD1), programmed death-ligand 1 (PD-L1), and the immunosuppressive IDO1 [148]. Additionally, myeloid-derived suppressor cells induced Gal-1 secretion by γδT cells, accelerating tumor growth by suppressing tumor-specific effector Tc cells and increasing the suppressive activity of Tregs [82]. It should be emphasized that many aspects of Gal-1-mediated signals have already been described in relation to tumor self-defense mechanisms. For a long time, it has been revealed that direct cell−cell communication between T cells and cancer cells in co-culture systems could induce T cell apoptosis *via* tumor-derived Gal-1 [149]. Adding recent findings to this topic, in colorectal carcinoma (CRC), both tumor and stroma-derived Gal-1 have been found to confer an immune privilege to the tumor by increasing the frequency and immunosuppressive activity of CD8^+^ Tregs in experimental models [150]. Corapi et al. further demonstrated an exciting scenario where the high levels of Gal-1 produced by prostate cancer (PCa) cells are not enough to evade immune attack; tumors require additional licensing by lymphocyte endogenous Gal-1 to be efficiently immune-suppressive [151].

Beyond its immunosuppressive effects, Gal-1 also impacts cancer cells themselves. Tumor-derived Gal-1 has been revealed to boost the proliferation and metastasis of gastric cancer cells *via* the neuropilin-1/c-JUN/Wee1 pathway [152] and enhance nanoclustering of the H-Ras proto-oncogene through direct interaction with the Ras binding domain of Raf [153]. Several proliferative signaling pathways are activated by Gal-1, including ERK and NF-ĸB, which drive the division of cancer cells [154], and the erythroid-2-related factor 2 (Nrf2), which is involved in cell survival and defense against oxidative stress in cancer cells [155]. Additionally, Gao et al. identified Gal-1 as a novel FOXP3-interacting protein, with nuclear Gal-1 reducing FOXP3’s tumor-suppressive effects in breast cancer [156]. Extracellularly present Gal-1 enhances tumor cell aggregation, possibly through interaction with mucin-1, and modulates cell–ECM connections in a concentration-dependent, biphasic manner, facilitating tumor cell migration and invasion [157]. Furthermore, stromal-secreted Gal-1 also promotes cancer stem cell-like properties and disease dissemination through SRY-box transcription factor 9 (SOX9) and β-catenin in this cancer [158]. It has also been found that tumor-activated cancer-associated fibroblasts (CAFs) overexpress Gal-1 and consequently release microvesicles containing increased levels of this protein. The uptake of Gal-1-enriched microvesicles by tumor cells then strongly affects cancer cell migration, suggesting a positive feedback loop between tumor cells and the TME [159]. Finally, emerging evidence highlights Gal-1’s role in epithelial-to-mesenchymal transition (EMT), a key process in tumor progression. For example, the depletion of TAMs leads to a decreased EMT signature in cancer cells, and Gal-1 has been identified as a crucial molecular factor in this process [160]. CAFs also express Gal-1, which induces EMT in gastric cancer cells, as evidenced by the upregulation of the mesenchymal marker vimentin and downregulation of the epithelial marker E-cadherin [161]. Gal-1 overexpression also fosters normal fibroblast transition into CAF, marked by increased production of TGF-β1 and alpha-smooth muscle actin (α-SMA) [162]. All these results indicate that Gal-1, both endogenously and exogenously – deriving from tumor cells, the tumor stroma, or tumor-infiltrating immune cell populations – supports cancer cell development and modulates the anti-tumor immune defense in several ways. Recognizing this complex, multifaceted nature of Gal-1’s effect on tumor growth, further research should be done with the aim of developing anti-cancer Gal-1 inhibitors [163, 164].

### Galectin-1 and cancer therapies

Gal-1 has been targeted by gene knock-down/silencing, blocking antibodies, therapeutic vaccines, or carbohydrate/non-carbohydrate inhibitors in several in vitro and in vivo model systems over the last two decades [163, 165–167]. These strategies have been employed to impair tumor growth and angiogenesis, as well as enhance the anti-tumor immune response [163, 168, 169]. For example, neutralization of tumor-derived Gal-1 with a thioredoxin-mouse Gal-1 therapeutic vaccine boosted the infiltration and cytotoxic activity of Tc cells against the tumor, promoted the normalization of the tumor vasculature, and decreased tumor burden. The authors suggested that vaccination eliciting an antibody response is a more practical strategy than Gal-1 knockdown in cancer patients since it’s cost-effective and can have less serious adverse effects [170]. Additionally, in a mouse glioma model, Gal-1-deficient tumor cells could be eradicated by host NK cells even before the initiation of an anti-tumor T cell response, suggesting that glioma cell-derived Gal-1 suppresses NK cell immune surveillance [171]. *LGALS1* repression also inhibited leukemia stem cell and leukemia cell proliferation and increased cell apoptosis in vitro. In vivo, it curbed acute myeloid leukemia progression and CD8^+^ T and NK cell count [172]. This study therefore highlighted that high *LGALS1* expression in leukemia stem cells is a significant risk factor for cancer development. All these findings propose that Gal-1 inhibition alone could be a potential cancer treatment strategy. However, it is more obvious to use it in combination with other existing cancer treatments for optimal efficacy.

Biological therapies against different types of tumors aim to reduce the side effects of systemic cancer treatment [173]. Among these, tumor immunotherapies seek to stimulate the immune system by regulating tumor cells and the TME, ultimately restoring T cell populations [174]. Costimulatory molecules on T cells serve as crucial checkpoints in regulating antigen-activated cell proliferation, functioning either as inhibitory [e.g., cytotoxic T lymphocyte antigen 4 (CTLA-4), programmed cell death protein 1 (PD-1), T-lymphocyte activation gene 3 (LAG-3), and T-cell immunoglobulin and mucin-domain containing-3 (TIM-3)] or stimulatory [e.g., CD28, CD40, immune co-stimulator (ICOS), and checkpoint molecules belonging to the tumor necrosis factor receptor superfamily] signals. Cancer cells exploit these inhibitory checkpoints by expressing their ligands that induce T cell apoptosis, thus avoiding specific adaptive immune attacks. This understanding led to the development of inhibitory immune checkpoint-blocking antibodies, such as anti-PD-1, anti-PD-L1, anti-CTLA-4, anti-LAG-3, and anti-TIM-3, which reverse inhibitory signals and enhance tumor-specific Tc cell proliferation [175, 176]. While immune checkpoint inhibitors (ICIs) undoubtedly revolutionized cancer treatments since they can significantly boost the immune responses against various tumors, their efficacy is limited by several factors. One major challenge is that ICIs can cause immune-related adverse events, which can be treated with various agents, depending on the symptoms [177]. Another challenge is that resistance to ICIs can also develop [178]. Related to this, the outcome of ICI therapy is influenced by the degree of T cell infiltration into the tumor and the Gal-1 expression level in the TME, as will be seen later. Therefore, using co-inhibitory immune checkpoints can help to defeat tumor resistance, and Gal-1 blockade fits well in this strategy. Here we report only a few exciting studies. For instance, in a glioblastoma multiforme (GBM) mouse model, intranasal Gal-1 silencing together with PD-1 blocking antibody had a synergistic effect, significantly increasing survival of tumor-bearing mice by reducing the number of Tregs and M2-type macrophages and increasing the number of Th and Tc cells [179]. Nambiar et al. reported an inverse correlation between tumor-expressed Gal-1 levels and the effectiveness of ICI treatment, as well as survival outcomes in patients with head and neck squamous cell carcinoma (HNSC). They highlighted that Gal-1 in the TME remodels the stromal endothelium by upregulating PD-L1 and Gal-9, which leads to T cell exclusion. Using genetic and pharmacological approaches to block Gal-1 led to increased intratumoral T cell infiltration and a better response to anti-PD1 therapy with or without radiotherapy [180]. In another study, the stromal accumulation of Gal-1 exhibited a significantly progressive upregulation, transitioning from low to intermediate grades and further escalating in high-grade PCa, where a small molecule inhibitor of Gal-1 (LLS30) potentiated antitumor activity of anti-PD1 in immunotherapy-resistant PCa in vivo [181]. Furthermore, Zheng et al. found that the KLF12−Gal-1 axis might serve as a novel therapeutic target, as decreased KLF12 and increased Gal-1 expression were associated with resistance to anti-PD-1 immunotherapy in various mouse tumor models. Consequently, increased KLF12 expression or the simultaneous use of Gal-1 inhibitors improved the anti-PD-1 response [182]. All these findings clearly demonstrate that high levels of Gal-1 could predict the negative response to ICI therapy and that combined blockade of ICIs and Gal-1 could improve therapy outcome.

Adoptive T cell transfer is an emerging immunotherapy approach in cancer treatment. This involves isolating γδ T cells from a patient’s own blood, expanding them ex vivo, and reinfusing them into the patient [183]. γδ T cells recognize and kill a wide range of tumor cells without the need for MHC molecules [184]. In a study, ex vivo-expanded γδ T cells, derived from tumor-infiltrating lymphocytes, demonstrated significant cytotoxicity against cervical cancer cells in vitro, which was further enhanced by the addition of an anti-Gal-1 antibody [185]. Furthermore, in vivo experiments showed that the combination therapy significantly suppressed tumor growth in mice compared to γδ T cells or anti-Gal-1 antibody alone. This suggests that anti-Gal-1 antibody treatment promotes synergistic anti-tumor effects of γδ T cells, therefore offering a more effective adoptive immunotherapy strategy for cervical cancer patients [185]. Autologous or allogeneic chimeric antigen receptor (CAR) T cells can also be infused into the patient after genetic modification to potentially cure leukemia and lymphomas [186]. Interestingly, Gal-1 has been identified as a critical factor used by myeloid leukemia cells to induce CAR down-regulation, resulting in impaired T-cell activation [187]. Therefore, combining CAR T cell therapy with Gal-1 blockade in these cases seems plausible [188].

Finally, several TF inhibitors are currently in clinical trials (e.g., SY-1365, a CDK7 inhibitor altering gene expression, including Runt-related transcription factor 1 (*RUNX1*) expression in advanced solid tumors) or in use (e.g., the estrogen receptor inhibitor Tamoxifen) to treat cancers [189]. These TFs are targeted to interfere with the hallmarks of cancers [190]. Fortunately, several of them have binding sites in the *LGALS1* promoter region (Fig. 2c, Table S2). Combining ICIs with drugs that reduce Gal-1 expression by targeting its activator TFs may offer another way to increase the efficacy of monotherapies.

In summary, all of the introduced combination therapeutic strategies hold great potential for improving the efficacy of existing cancer treatments. However, several challenges remain to be addressed, including the selection and prioritization of regimens based on pre-clinical and clinical data, the development of predictive biomarkers, and the evaluation and management of adverse events.

## Limitations of in vitro and in vivo studies in galectin-1 research

The main challenge in studying cellular responses of Gal-1 (and other galectins) is that it can bind a wide range of glycoconjugates present in cells and in ECMs. Therefore, when it is released from one type of cell in the body, it can bind to a variety of cells, depending on several factors: (1) It can bind back to the source cell; (2) It can be “trapped” in the ECM; (3) It can affect different types of neighboring responsive cells in the same tissue; (4) It can travel far from the source cell packed into EVs and act on distant cells. This is obviously influenced by several factors, like the mode of cellular release (soluble or EV-packed), the oxidative state of the environment, the abundance of its glycoepitopes, the local concentration of Gal-1, the local concentration of galectins competing for the same ligands, or, the opposite, forming galectin hybrid heterodimers, thereby tuning the galectin-glycan lattice network. Therefore, it is increasingly evident that the outcomes of those studies in which cellular responses to Gal-1 have been examined in cell cultures, similar to methods used to study other soluble proteins with unique interacting partners, do not always confirm or reflect the in vivo actions of Gal-1. On the other hand, regarding the mentioned complex and pleiotropic Gal-1 effector mechanisms, the observed reactions in whole animal experiments may not be properly connected to the outcomes of the cultured cell-based results. Therefore, we have to be critical in interpreting these studies while the authors often have not mentioned the tissues and cell types in mice that the injected protein targeted, as they have done in the case of in vitro experiments. Furthermore, in light of the impacts of endogenously functioning Gal-1, as it has been discussed, we must question the accuracy of studies that only address the reactions to exogenously provided recombinant Gal-1 without considering the potential impact of the altered intracellular Gal-1 signalization. The question is whether the intracellularly acting Gal-1 also modifies the phenotypes attributed to the externally administered Gal-1. Non-separable intracellular and extracellular actions of Gal-1 can be the partial cause of sometimes contradictory and misleading findings reported in the literature. These limitations have already been identified by others as well, and fortunately for the field, a growing number of studies have examined the roles of endogenous galectins by investigating mice that have specific galectin knocked-out genes in specific cells.

## Concluding remarks and future perspectives

**Fig. 6:**
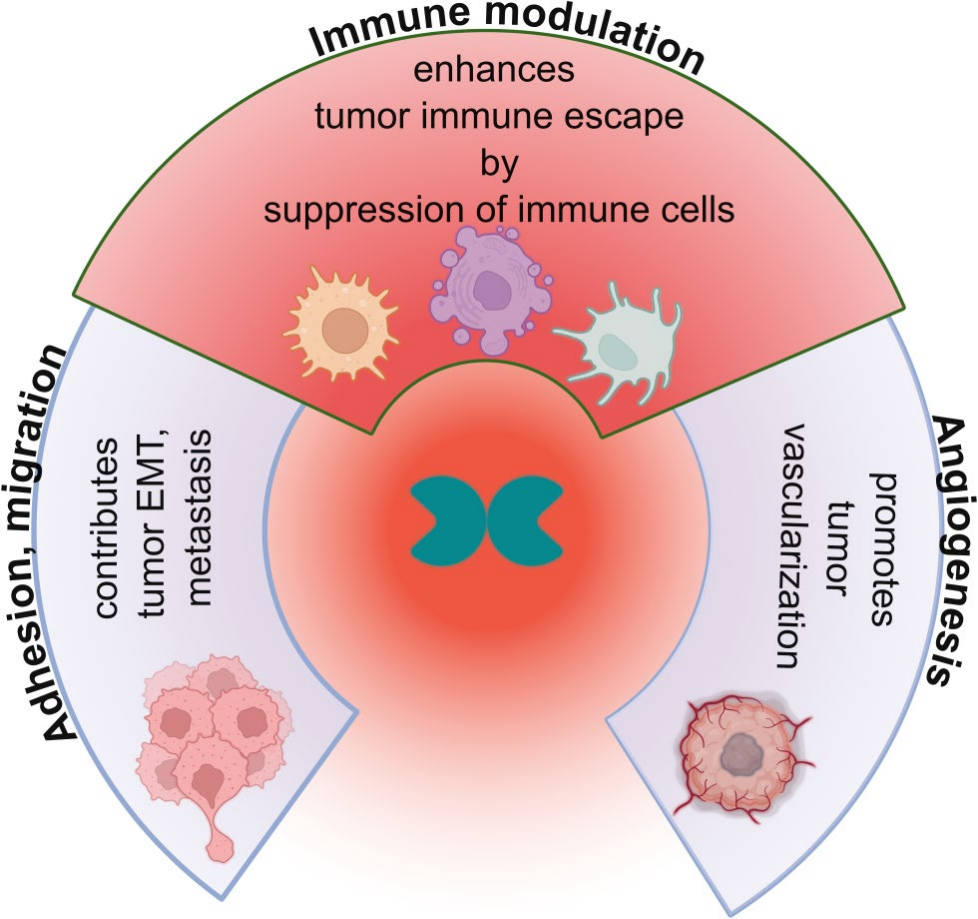
The role of galectin-1 in cancer formation. Selected cancer hallmarks reflecting the main physiological functions of Gal-1 are illustrated. It promotes angiogenesis by inducing the proliferation of endothelial cells *via* interacting with e.g., VEGF-2 [197]. Gal-1 also facilitates immune escape by suppressing the immune response, inducing the apoptosis of activated T cells, reducing inflammatory responses in macrophages, promoting macrophage polarization towards the M2 phenotype, and inhibiting the function of NK cells. Additionally, Gal-1 regulates cell adhesion and migration by modulating cell−cell and cell−ECM interactions *via* integrins and ECM components [5]. Gal-1 also promotes the EMT process by enhancing the migration and invasion capabilities of cancer cells, which in turn, facilitate metastasis. ECM, extracellular matrix; NK, natural killer; VEGF-2, vascular endothelial growth factor 2.

In addition to the outlined limitations, it can be stated that the multifaceted immunosuppressive properties of Gal-1 – beneficial in immune-privileged sites and for resolving inflammation, but detrimental in tumor immune evasion (Fig. 6) – entitle this lectin to be classified as a glyco-checkpoint molecule and an oncogene and make it an attractive target for enhancing existing cancer therapies. Inhibiting Gal-1 can restore anti-tumor immune responses, particularly those of tumor-specific T cells, thereby improving the efficacy of chemo- and immunotherapies while reducing tumor resistance. Early research results are encouraging, showing improved therapeutic outcomes with manageable safety profiles. However, determining the most efficient method for Gal-1 blockade and integrating it with the most potent therapy within the framework of precision cancer medicine remains a challenge. The optimal combination will probably depend on the tumor’s type, grade, and molecular profile. Nevertheless, caution is required regarding short- and long-term side effects of Gal-1 inhibition, given its wide range of target cells and receptors. This can be partially overcome by delivering Gal-1 inhibitors to the tumor site. Finally, but just as importantly, Gal-1 can be targeted in conjunction with other galectin family members in cases where its blockade proves insufficient.

## Electronic supplementary material

Below is the link to the electronic supplementary material.

Supplementary Material 1

Supplementary Material 2

## Data Availability

Table S1 contains the full datasets of *LGALS1* expression in humans, derived from bulk-tissue and single-cell RNA-seq, as well as RNA-seq of immune cells. Data were downloaded from the Human Protein Atlas on 13-05-2024: consensus bulk RNA-seq data of HPA and GTEx projects, single-cell RNA-seq data from various external sources, and immune cell gene data. Table S2 contains the complete list of transcription factors of *LGALS1* in the 1000 bp 5’ upstream region of the gene. The list is the cross-section of datasets from JASPAR 2024 and TFLink open-access databases downloaded on 04-04-2024, with a minimum score of 240.

## Abbreviations

α-SMA: Alpha-smooth muscle actin
ACOD1: Cis-aconitate decarboxylase
AP-1: Activator protein 1
APC: Antigen-presenting cell
β-Gal: β-galactoside
Bcl-2: B-cell lymphoma 2 protein
CAF: Cancer-associated fibroblast
C2GNT: Core 2 β-1,6-N-acetylglucosaminyltransferase
CCL2: C-C motif chemokine ligand 2
CAR: Chimeric antigen receptor
CESC: Cervical squamous cell carcinoma
CRD: Carbohydrate recognition domains
cHL: Classical Hodgkin lymphoma
CTLA-4: Cytotoxic T-lymphocyte antigen 4
CtxB: Cholera toxin subunit B
COAD: Colon adenocarcinoma
CXCL5: C-X-C motif chemokine ligand 5
OV: Ovarian serous cystadenocarcinoma
DC: Dendritic cells
DLBC: Diffuse large B cell lymphoma
ECM: Extracellular matrix
EMT: Epithelial-to-mesenchymal transition
ESCA: Esophageal carcinoma
ESR1: Estrogen receptor 1
ESR2: Estrogen receptor 2
ERK: Extracellular signal-regulated kinase
ERRα: Estrogen-related receptor alpha
EV: Extracellular vesicle
EVT: Extravillous trophoblast
Fas: Fas cell surface death receptor
FasL: Fas cell surface death receptor ligand
FOS: Fos proto-oncogen
FGF: Fibroblast growth factor
FGFR1: Fibroblast growth factor receptor 1
FOXP3: Forkhead box P3
Gal-1: Galectin-1
Gal-9: Galectin-9
GBM: Glioblastoma multiforme
HIF-1α: Hypoxia-inducible factor 1-alpha
HNSC: Head and neck squamous cell carcinoma
HRE: Hypoxia-response elements
HSC: Hematopoietic stem cell
IDO: Indoleamine-pyrrole 2,3-dioxygenase
ICI: Immune checkpoint inhibitors
ICOS: Immune co-stimulator
IFN-γ: Interferon gamma
IL: Interleukin
JAK3: Janus kinase 3
JUN: Jun proto-oncogen
KIRC: Kidney renal clear cell carcinoma
KIRP: Kidney renal papillary cell carcinoma
KLF12: Krüppel-like factor 12
LAG-3: T-lymphocyte-activation gene 3
MAPK: Mitogen-activated protein kinase
MHC: Major histocompatibility complex
MSC: Mesenchymal stem cell
Nrf2: Erythroid-2-related factor 2
NEU1: Neuraminidase 1
NF-ĸB: Nuclear factor kappa B
NK: Natural killer
NSCLC: Non-small cell lung cancer
PAAD: Pancreatic adenocarcinoma
PBMC: Peripheral blood mononuclear cell
PCa: Prostate cancer
PD-1: Programmed cell death protein 1
PD-L1: Programmed death-ligand 1
PGF: Placental growth factor
PGR: Progesterone receptor
PRAD: Prostate adenocarcinoma
pre-BCR: Pre-B cell receptor
READ: Rectum adenocarcinoma
RORC2: Retinoic acid receptor-related orphan receptor C2
RUNX1: Runt-related transcription factor 1
SKCM: Skin cutaneous melanoma
SOX9: SRY-Box Transcription Factor 9
ST3GAL6: ST3 beta-galactoside alpha-2,3-sialyltransferase 6
ST6GAL1: ST6 beta-galactoside alpha-2,6-sialyltransferase 1
STAD: Stomach adenocarcinoma
STAT: Signal transducer and activator of transcription
TAM: Tumor-associated macrophage
Tc: Cytotoxic T cell
TCR: T cell receptor
TF: Transcription factor
TGF-β: Transforming growth factor-β
Th: Helper T cell
THYM: Thymomas and thymic carcinomas
TIM-3: T-cell immunoglobulin and mucin-domain containing-3
TME: Tumor microenvironment
Tr1: Type 1 regulatory cell
Treg: Regulatory T cell
UCEC: Uterine corpus endometrial carcinoma
VEGF: Vascular endothelial growth factor
VEGFR: Vascular endothelial growth factor receptor
VSTM1: V-set and transmembrane domain-containing 1
